# Pleiotropic Effects of Levofloxacin, Fluoroquinolone Antibiotics, against Influenza Virus-Induced Lung Injury

**DOI:** 10.1371/journal.pone.0130248

**Published:** 2015-06-18

**Authors:** Yuki Enoki, Yu Ishima, Ryota Tanaka, Keizo Sato, Kazuhiko Kimachi, Tatsuya Shirai, Hiroshi Watanabe, Victor T. G. Chuang, Yukio Fujiwara, Motohiro Takeya, Masaki Otagiri, Toru Maruyama

**Affiliations:** 1 Department of Biopharmaceutics, Graduate School of Pharmaceutical Sciences, Kumamoto University, 5–1 Oe-Honmachi, Chuo-ku, Kumamoto, 862–0973, Japan; 2 Center for Clinical Pharmaceutical Sciences, School of Pharmacy, Kumamoto University, 5–1 Oe-Honmachi, Chuo-ku, Kumamoto, 862–0973, Japan; 3 Department of Clinical Biochemistry, School of Pharmaceutical Sciences, Kyushu University of Health and Welfare, Yoshino-Machi, Nobeoka, Japan; 4 The Chemo-Sero-Therapeutic Research Institute (KAKETSUKEN), 1-6-1 Okubo, Kita-ku, Kumamoto-shi, Kumamoto, 860–8568, Japan; 5 School of Pharmacy, Faculty of Health Sciences, Curtin Health Innovation Research Institute, Curtin University, GPO Box U1987, Perth, 6845, Western Australia; 6 Graduate School of Medical Sciences, Faculty of Life Sciences, Kumamoto University, 1-1-1 Honjo, Chuo-ku, Kumamoto, 860–0811, Japan; 7 Faculty of Pharmaceutical Sciences, Sojo University, 1-22-4 Ikeda, Nishi-ku, Kumamoto, 860–0082, Japan; 8 DDS Research Institute, Sojo University, 1-22-4 Ikeda, Nishi-ku, Kumamoto, 860–0082, Japan; Indiana University, UNITED STATES

## Abstract

Reactive oxygen species (ROS) and nitric oxide (NO) are major pathogenic molecules produced during viral lung infections, including influenza. While fluoroquinolones are widely used as antimicrobial agents for treating a variety of bacterial infections, including secondary infections associated with the influenza virus, it has been reported that they also function as anti-oxidants against ROS and as a NO regulator. Therefore, we hypothesized that levofloxacin (LVFX), one of the most frequently used fluoroquinolone derivatives, may attenuate pulmonary injuries associated with influenza virus infections by inhibiting the production of ROS species such as hydroxyl radicals and neutrophil-derived NO that is produced during an influenza viral infection. The therapeutic impact of LVFX was examined in a PR8 (H1N1) influenza virus-induced lung injury mouse model. ESR spin-trapping experiments indicated that LVFX showed scavenging activity against neutrophil-derived hydroxyl radicals. LVFX markedly improved the survival rate of mice that were infected with the influenza virus in a dose-dependent manner. In addition, the LVFX treatment resulted in a dose-dependent decrease in the level of 8-hydroxy-2’-deoxyguanosine (a marker of oxidative stress) and nitrotyrosine (a nitrative marker) in the lungs of virus-infected mice, and the nitrite/nitrate ratio (NO metabolites) and IFN-γ in BALF. These results indicate that LVFX may be of substantial benefit in the treatment of various acute inflammatory disorders such as influenza virus-induced pneumonia, by inhibiting inflammatory cell responses and suppressing the overproduction of NO in the lungs.

## Introduction

Recent evidence derived from both basic and clinical studies suggest that a pleiotropic effect, i.e., an effect other than that for which the agent was specifically intended, may be of value in the treatment of a variety of diseases. For example, erythromycin, a macrolide antibiotic, has been reported to be effective for the treatment of chronic airway diseases, such as diffuse bronchiolitis, bronchial asthma, and chronic sinusitis. These therapeutic benefits of erythromycin extend beyond its antibiotic properties and the mechanism responsible for such pleiotropic effects includes the inhibition of neutrophil migration [1, 2], the suppression of IL-8 production [3] and Cl-secretion inhibitory action [4].

Influenza virus infections are a serious public health problem that results in severe illness and death in high risk populations. The current therapeutic strategies for combating influenza virus infections involve directly targeting the influenza virus itself [5, 6]. However, it is well known that such infections often induce a lethal acute lung injury, which largely contributes to the overproduction of reactive oxygen species (ROS) and reactive nitrogen oxide species (RNOS) induced by complicated interactions that occur between the virus and host, including immunologic effects of the host [7]. In addition, it has recently been reported that Nox2, which is expressed at high levels in inflammatory cells, including neutrophils, and serves as a major source of ROS in infectious diseases, is produced at excessively high levels during influenza infections, leading to a deterioration in the overall health of the patient, and is considered to be a key molecule in the development of influenza virus-induced pneumonia [8]. Therefore, it has been proposed that scavengers of ROS or RNOS could represent a potentially new therapeutic strategy for the treatment of influenza virus-induced pneumonia [9–11]. In fact, a number of compounds, including N-acetyl-cysteine, superoxide dismutase (SOD), catalase, thioredoxin, N^G^-Monomethyl-L-arginine that possess scavenger or inhibitor activity against free radicals, but do not show an antivirus effect, have been reported to be effective in treating mice that had been infected with the influenza virus [8, 11–13]. Since the pulmonary damage induced by an influenza virus infection increases the risk of a secondary infection by bacteria, such as a pneumococcus, an anti-oxidant therapy is also important from the point of view of suppressing secondary bacterial infections [14].

Fluoroquinolones (FQs), antimicrobial drugs with a broad spectrum of antibacterial activity are frequently used in the treatment of various infections. Among these drugs, respiratory quinolones such as tosufloxacin, sparfloxacin and levofloxacin (LVFX) have been found to be particularly effective in treating most pneumococci, which are associated with respiratory tract infections [15]. These respiratory quinolones have been demonstrated to have antibacterial effects against secondary infections after the induction of pulmonary damage caused by an influenza infection. In addition to antibacterial activity, it has been reported that FQs also function as an immunomodulator [16, 17], an anti-oxidant agent [18] and a nitric oxide (NO) regulator [19]. These findings led us to conclude that FQs may have the potential to inhibit influenza virus-induced pneumonia via its pleiotropic effects, including its anti-oxidative and NO inhibitory properties. If that is the case, FQ would be expected to be, not only effective for the treatment of pulmonary damage associated with influenza virus infections, but also might inhibit the development of secondary infections caused by bacteria.

The purpose of this study was to investigate the pleiotropic effects of LVFX, one of the most frequently used FQs, and to examine its therapeutic impact on the acute lung damage associated influenza virus infections using the PR8 (H1N1) influenza virus. To elucidate the mechanism responsible for the cytoprotective effect of LVFX on pulmonary damage, its anti-oxidant activities against ROS and NO *in vitro* and *in vivo* were evaluated using electron spin resonance spin trapping and immunohistochemical techniques.

## Materials and Methods

### Animal ethics statement

All animal experiments were approved by the experimental animal ethics committee at the Kumamoto University. All animal experiments were conducted in accordance with the guidelines of Kumamoto University for the care and use of laboratory animals (B 26–058).

### Materials

5, 5-dimethyl-1-pyrroline-N-oxide (DMPO) was purchased from Alexis Biochemicals (Lausen, Switzerland). Ciprofloxacin (CPFX), norfloxacin (NRFX), pazfloxacin (PZFX), levofloxacin (LVFX), lomefloxacin (LFLX) and 1, 3-dimethylthiourea (DMTU) were purchased from Tokyo Chemical Industry Co., Ltd (Tokyo, Japan). Diethylenetriaminepentaacetic acid (DTPA), NaNO_2_, NaNO_3_ and dextran were purchased from Nacalai Tesque (Kyoto, Japan). Ficoll-Paque was purchased from GE Healthcare (Tokyo, Japan), and phorbol 12-myristate 13-acetate (PMA) was purchased from Wako Pure Chemical Industries, Ltd (Osaka, Japan). Mayer’s hematoxylin and eosin alcohol solutions were purchased from Muto Pure Chemicals (Tokyo, Japan). Superoxide dismutase from bovine erythrocytes was purchased from Sigma (Tokyo, Japan).

### Influenza virus and cell culture

The influenza virus (PR8 [H1N1]) used in this study was generously provided by KAKETSUKEN (Kumamoto, Japan). H1N1 influenza is a common seasonal type of influenza virus, and the H1N1 mouse-adapted influenza virus A/PR/8/34 (PR8) is a commonly used model for an H1N1 infection. Madin-Darby Canine Kidney (MDCK) cell (ATCC, Rockville, MD, USA) was cultured in Dulbecco's modified Eagle medium (DMEM, Life technologies Co., Ltd, California, USA) supplemented with 10% heat inactivated fetal bovine serum (Hyclone Laboratories, Logan UT, USA) and 100 units/ml penicillin and 100 μg/ml streptomycin (Life technologies Co., Ltd, California, USA), at 37°C in a humidified incubator with 5% CO_2_.

### Mice

Five week old, specific pathogen-free male ICR mice were purchased from Kyudo Co., Ltd (Saga, Japan). The animals were bred at the Center for Animal Resources and Development and housed at 22 ± 2°C on a 12 h day/night cycle.

### Measurement of scavenging activity against OH radicals

OH radicals were spin-trapped by DMPO and the scavenging activity of each of the FQs (CPFX, NRFX, PZFX, LVFX and LFLX) was calculated based on the relative intensity of the peak of the ESR signal for the DMPO-OH radical adduct (DMPO-OH). Reaction mixtures, which contained 500 μM H_2_O_2_, 100 μM DTPA and 4.5 mM DMPO, were incubated with each FQ and were immediately transferred to an ESR flat cell and irradiated at 254 nm for 30 s. After UV-irradiation, the ESR flat cells were immediately placed in a JES-TE 200 ESR spectrometer (JEOL, Tokyo, Japan), and ESR spectra were recorded at 25°C under the following conditions: modulation frequency, 100 kHz; microwave frequency, 9.43 GHz; microwave power, 40 mW; scanning field, 335.2 ± 5 mT; sweep time, 2 min; field modulation width, 0.25 mT; receiver gain, 1000; and time count, 0.3 s.

### Isolation of polymorphonuclear neutrophils

Whole blood was obtained from 10 mice. Heparinized blood was mixed with an equal volume of 3% dextran in 0.9% NaCl. After 30 min of gravity sedimentation, the upper layer, containing leukocytes, was collected and centrifuged at 620 × g for 10 min. The pellet was resuspended in 0.9% NaCl and underlaid with Ficoll-Paque. After centrifugation for 30 min at 1,490 × g, the mononuclear cell layer was isolated and contaminating red blood cells were removed by hypotonic lysis, After centrifugation for 10 min at 760 × g (two times), the pellet was resuspended in hanks balanced saline solution (HBSS, Sigma, Tokyo, Japan).

### Measurement of scavenging activity against neutrophil-derived ROS

The scavenging activity of LVFX against ROS released from neutrophils was determined using an ESR spin trapping method with DMPO. The neutrophils (1.0 × 10^5^ cells/ml) were incubated with LVFX and 100 ng/ml of PMA at 37°C for 7 min to activate the cells and allow the generation of ROS. After the incubation, DMPO (22.5 mM final concentration) was added to this reaction mixture. ESR spectra were recorded at 25°C in a JES-TE-200 spectrometer after 2 min under the following conditions: modulation frequency, 100 kHz; microwave frequency, 9.43 GHz; microwave power, 40 mW; scanning field, 335.2 ± 5 mT; sweep time, 2 min; field modulation width, 0.25 mT; receiver gain, 1000; and time count, 0.3 s.

### Production of influenza virus-induced lung injury model mice

Influenza virus-induced lung injury model mice were produced by the intratracheal administration of influenza virus suspended LB medium (20 μl/mice) under anesthesia with chloral hydrate (500 mg/kg) on day 0. ‎It should be noted that anesthesia with chloral hydrate did not affect the initiation and progression of influenza virus-induced lung injury and mortality (S1 Fig). The virus infection dose was determined by mice mortality by inoculating mice with serial dilutions of the virus suspension (data not shown). LVFX (25 or 100 mg/kg) was administrated intraperitoneally once per day, starting on day 2 until day 6 and the survival rate and body weight of animals were recorded daily until day 14 (S2 Fig). Phosphate buffered saline (PBS) was used as vehicle solution for LVFX and the vehicle group mice were administered PBS intraperitoneally. For humane endpoints, the mice that showed evidence of distress (ruffled fur, slowed respiration, no response to cage tapping) or a 30% weight loss were euthanized by an intraperitoneal injection of pentobarbital sodium (100 mg/kg) and recorded as a mortality.

### HE staining

At 7 days after the virus infection, the mice were sacrificed and whole lungs were removed under anesthesia with diethyl ether. The removed lungs were fixed in 4% buffered paraformaldehyde and then embedded in paraffin, which were then cut into 4-μm-thick sections. Sections were stained first with Mayer’s hematoxylin and then with a 1% eosin alcohol solution. HE staining samples were mounted with malinol and inspected with the aid of a microscope (Keyence, BZ-8000, Osaka, Japan). The histological severity of lung was blindly scored followed by previous reports partly modified [20–22].

### Measurement of virus titer

Lungs from mice on day 7 after virus infection were collected and homogenized in 2 ml of RPMI 1640 medium. Virus titers were determined by a plaque assay on MDCK cells. MDCK cells were seeded on a 24 well culture plate and cultured until reaching confluency. Cells were washed with PBS (-), and then 0.1-ml amounts of each dilution were inoculated to cultured cell followed by incubation for 1 h at 34°C. DMEM (0.5 ml) containing 100 U/mL penicillin, 100 μg/ml streptomycin and 0.5 μg/ml fungizone was added to each well and incubated at 37°C for 3 days in a humidified incubator with 5% CO_2_. The cytopathological effect was calculated and 50% tissue culture infectious dose (TCID_50_) was calculated using the formula developed by Reed and Muench [23].

### Immunostaining of lung tissue

On day 7 after virus infection, the mice were sacrificed and whole lungs were removed under anesthesia with diethyl ether. The removed lungs were fixed in 4% buffered paraformaldehyde and then embedded in paraffin, which were then cut into 4-μm-thick sections. After antigen activation with immunosaver (nisshin EM Co., Ltd, Tokyo, Japan), a solution containing 50 mM Tris/HCl + 0.1% Tween-20 (T-TB) was used to solubilize the lung slices, followed by blocking with Block Ace (Dainippon Pharmaceutics, Osaka, Japan) at room temperature for 15 min. The primary antibody reaction was then conducted below 4°C overnight. The primary antibody containing 8-hydroxy-2’-deoxygenase (8-OH-dG) [15A3] (Santa Cruz Biotechnology Inc, cat#: sc-66036, Santa Cruz, USA) and nitrotyrosine (NO_2_-Tyr) (Millipore, cat#: AB5411, Billerica, USA) was diluted 50 times before use. The lung slices were then washed with 50 mM Tris/HCl (TB) and 0.1% Tween-TB, followed by the secondary antibody reaction at 25°C for 1.5 h. For the secondary antibody of NO_2_-Tyr and 8-OH-dG, Alexa Fluor 546 goat anti-mouse IgG (H+L) (Invitrogen, Eugene, USA) and Alexa Fluor 488 goat anti-mouse IgG (H+L) (Invitrogen, Eugene, USA) were diluted 200 times, respectively. After the reaction, the slide was observed by microscopy (Keyence, BZ-8000 microscope, Osaka, Japan). Image analyses of the extent and intensity of 8-OH-dG and NO_2_-Tyr staining were also performed using the imageJ software.

### d-ROMs test

Whole blood was obtained from mice under anesthesia with diethyl ether on day 7 after administration of the influenza virus. Whole blood was centrifuged at 1,000 × g for 10 min and the resulting serum was used for the assay. Derivatives of reactive oxidative metabolites were assayed using FREE carpe diem (Diacron International s. r. l., Grosseto, Italy) following the manufacturer’s recommended protocols.

### Measurement of NO and IFN-γ in BALF

The mice were anesthetized with diethyl ether, the chests were opened on day 7 after administration of the influenza virus. BALF was collected by cannulating the trachea with 1 ml of sterile PBS containing heparin. About 1.8 ml of BALF was routinely recovered from each animal after 2 lavages. The BALF was centrifuged at 4,100 × g for 5 min at 4°C to separate the cells in the BALF from the liquid. The supernatant (10 μl) was used in the assay. Nitrate and nitrite (NOx) were measured using an EiCOM ENO-20 NOx analyzer (EiCOM, Kyoto, Japan). The mobile phase was NOCARA (EiCOM, Kyoto, Japan) and reaction solution was comprised of Griess reagents. Equal concentrations of a mixture of NaNO_2_ and NaNO_3_ were used to prepare a standard curve. Serum IFN-γ was measured using an ELISA MAX Deluxe Set (Biolegend, San Diego, USA) following the manufacturer’s protocols.

### Data analysis

Data are the mean ± SD in each experiment. Significant differences among each group were examined using a one-way of analysis of variance (ANOVA) followed by Tukey multiple comparison. A probability value of p< 0.05 was considered to indicate statistical significant.

## Results

### Anti-oxidative effect of LVFX (*In vitro*)

**Fig 1:**
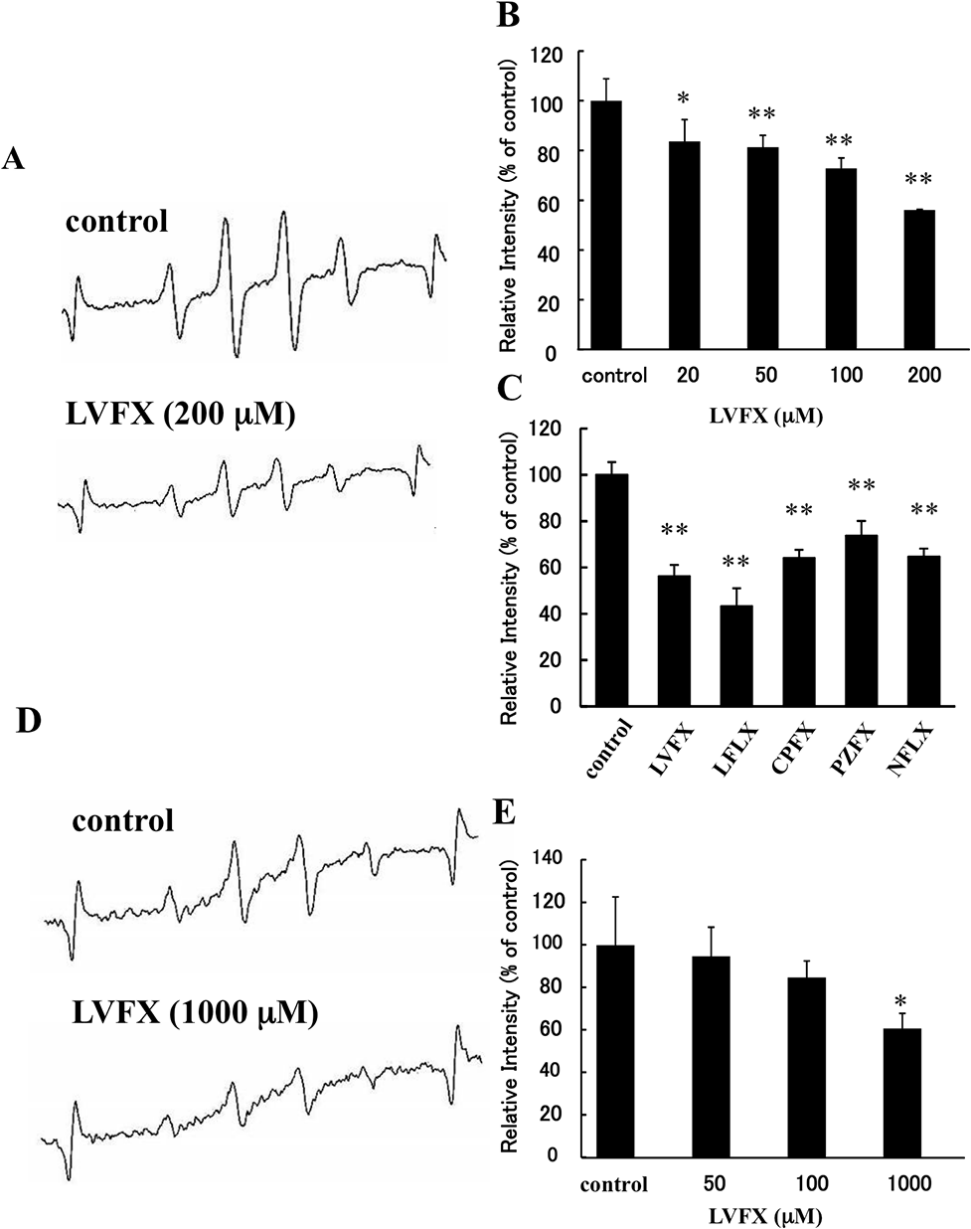
Measurement of anti-oxidative effects of LVFX using EPR spectroscopy. The scavenging activities of LVFX against ROS derived from the UV/H_2_O_2_ system or neutrophils stimulated with 100 ng/ml PMA were determined using ESR spin trapping with DMPO. (A) The reaction mixtures, which contained 500 μM H_2_O_2_, 100 μM DTPA and 4.5 mM DMPO, were incubated with each FQ and were immediately transferred to an ESR flat cell and irradiated at 254 nm for 30 s. After UV-irradiation, the ESR flat cells immediately placed in a JES-TE 200 ESR spectrometer. ESR spectrum of DMPO spin adducts of OH radicals and (B) the quantitation of the OH radicals concentration were shown. (C) The scavenging activity of 200 μM FQs; lomefloxacin (LFLX), levofloxacin (LVFX), Ciprofloxacin (CPFX), pazfloxacin (PZFX), and norfloxacin (NRFX) against OH radicals. (D) ESR spectrum of DMPO spin adducts ROS derived from neutrophils containing OH radicals, and (E) the quantitation of the ROS concentration were shown. Each bar represents the mean ± SD (n = 3). *p<0.05, **p<0.01 vs control.

We first examined the scavenging activity of LVFX for OH radicals, a highly toxic type of ROS, using an ESR spin-trapping method. As shown in Fig 1A and 1B, the ESR signal intensities produced by a H_2_O_2_/UV system indicate that LVFX scavenges radicals in a dose-dependent manner. Similar to LVFX, four other FQs, LFLX, CPFX, PZFX, NFLX, also showed scavenging activity against OH radicals (Fig 1C), suggesting that the OH radical scavenging ability of FQs might be dependent on the presence of a 4-quinolone ring in the molecule, the basic chemical structural unit of FQ. We also examined the ROS scavenging activity of LVFX derived from activated neutrophils. As shown in Fig 1D and 1E, LVFX showed scavenging activities against ROS derived from neutrophils and the scavenging was dose-dependent.

### Effect of LVFX on the survival of influenza virus-induced lung injury mice

**Fig 2:**
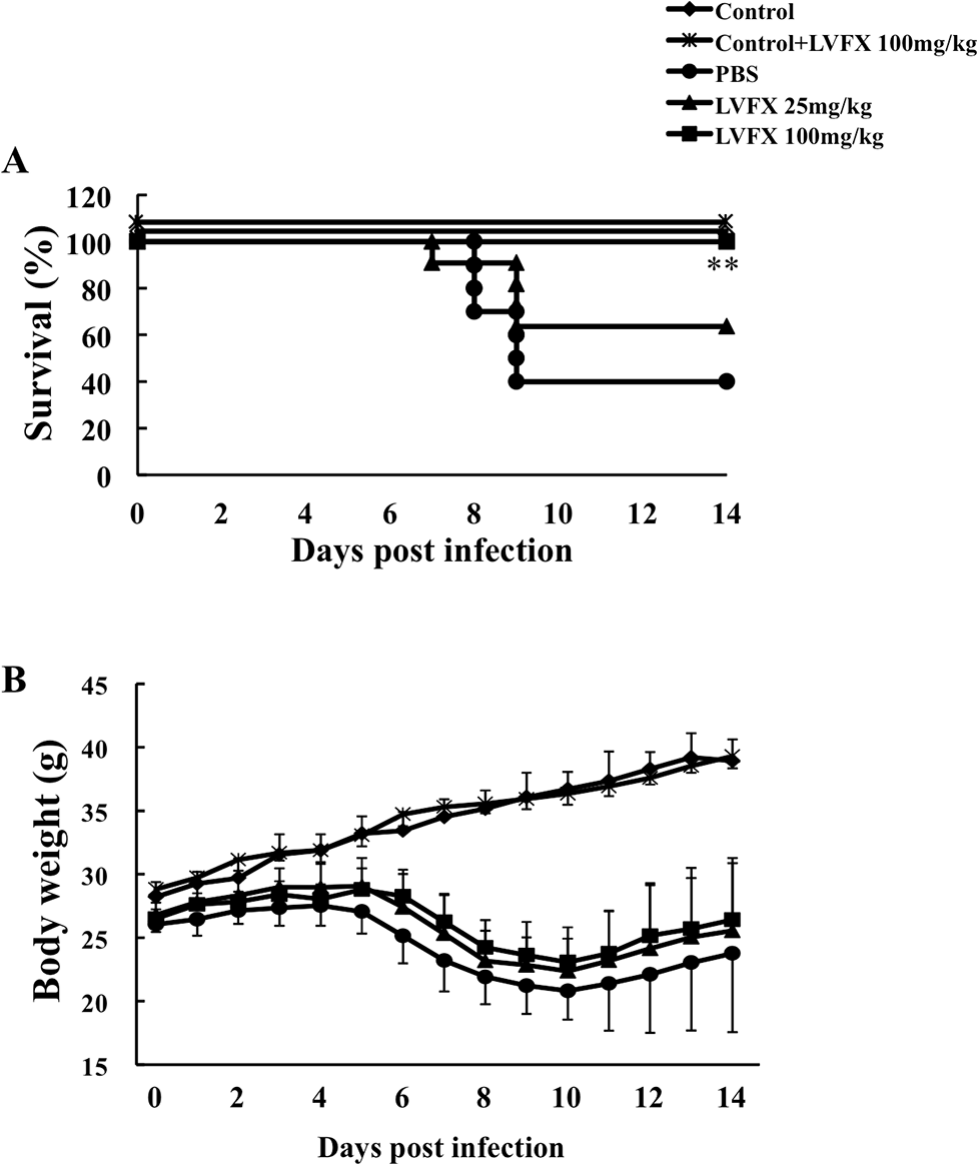
Therapeutic effect of LVFX on influenza virus-infected mice. Influenza virus infected mice were produced by the intratracheal administration of influenza virus under anesthesia on day 0. After infection, the mice were treated with LVFX on day 2 after infection by means of an intraperitoneal injection of LVFX. (A) Survival curves for control (n = 3), 100 mg/kg LVFX (not infected, n = 3), PBS (n = 10), 25 mg/kg LVFX (n = 10) and 100 mg/kg LVFX (n = 10) treatment during an influenza virus infection. (B) Weight loss during an influenza virus infection. Each bar represents the mean ± SD. **p<0.01 vs vehicle. Experiment was repeated three times.

We next examined the therapeutic impact of LVFX against the lethal effect of influenza virus induced pneumonia in mice. In this experiment, we monitored the time course for the survival rate and the body weight of mice after the virus infection with or without an intraperitoneal administration of LVFX (25 and 100 mg/kg/day) between day 2 and day 6. As shown in Fig 2A, 60% of the infected mice died within 9 days after being infected, however, LVFX markedly reduced this lethal effect and the effect was dose dependent. The survival rate of mice that had been treated with LVFX at a dose of 25 or 100 mg/kg was 60 or 100%, respectively. As shown in Fig 2B and S3 Fig there was no significant difference in influenza virus induced body weight loss between the LVFX and saline treated groups.

### Histopathological analysis of lung tissue

**Fig 3:**
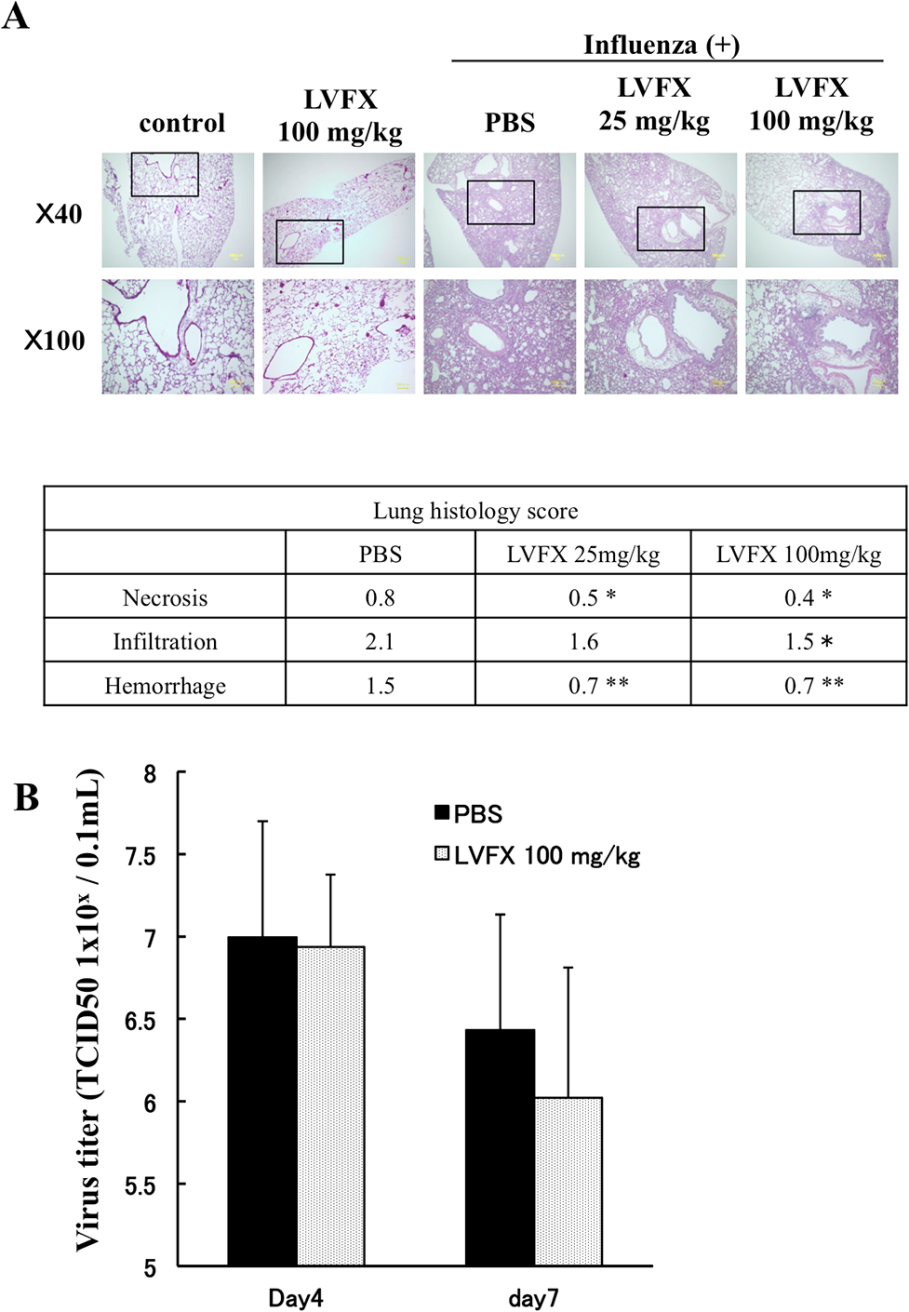
The effect of LVFX on lung damage and viral titer in influenza virus-infected mice. (A) Section of lung tissue were prepared at the day 7 after influenza virus infection, and subjected to histopathological examination with HE staining (upper). The alveolar space was observed in lungs from control mice, however the influenza virus infection caused a marked increase in the infiltration of inflammatory cells into the alveoli or peribronchial areas, and the alveolar space was completely filled with these cells. Treatment with LVFX at a dose of 100 mg/kg decreased the overall infiltration of inflammatory cells. The histopathological severity of lung section was determined and average score showed in lower of HE staining image. Histological score shown PBS (n = 4), 25 mg/kg LVFX (n = 5) and 100 mg/kg LVFX (n = 5). (B) The effect of LVFX on viral load at day 4 and 7 were determined using a plaque forming assay. Each bar represents the mean ± SD (n = 3–5). *p<0.05, **p<0.01 vs PBS.

To confirm whether the therapeutic effect of LVFX shown in Fig 2 was due to the inhibition of pulmonary damage induced caused by the influenza infection, the protective effect of LVFX was examined histopathologically (S4 Fig), and the severity of histological changes was graded according to a semiquantitative scoring system. As shown in Fig 3A (upper), HE staining data showed that the influenza virus caused severe lung damage, with an excessive infiltration of inflammatory cells into alveoli or the bronchial pathway. The objective scoring for the lung tissue sections indicate that a PR8 infection cause inflammatory infiltration, hemorrhage and mild necrosis. However, the LVFX (100 mg/kg/day) treatment significantly suppressed these observations compared with PBS treatment group (Fig 3A lower).

### Effect of LVFX on influenza virus titer in mice

To confirm the effect of LVFX on the replication of the influenza virus, we quantified the virus titer in lung homogenates of mice obtained on day 4 and 7 after the influenza virus treatment with or without the administration of LVFX (100 mg/kg/day). As shown in Fig 3B, the LVFX treatment had little effect on the virus titer of the infected mice at both day 4 and 7 (Fig 3B). In addition, it is unlikely that a secondary bacterial infection would have developed in the mouse model used in this study, because, when homogenized lung tissues from influenza virus infected mice were subjected to a colony-formation assay on trypticase soy agar, no bacteria were detected (data not shown). These results suggest that the suppression of lung damage by LVFX does not involve a reduction in virus replication or the inhibition of secondary bacterial infections.

### Anti-oxidative effect of LVFX on influenza virus-induced lung injury

**Fig 4:**
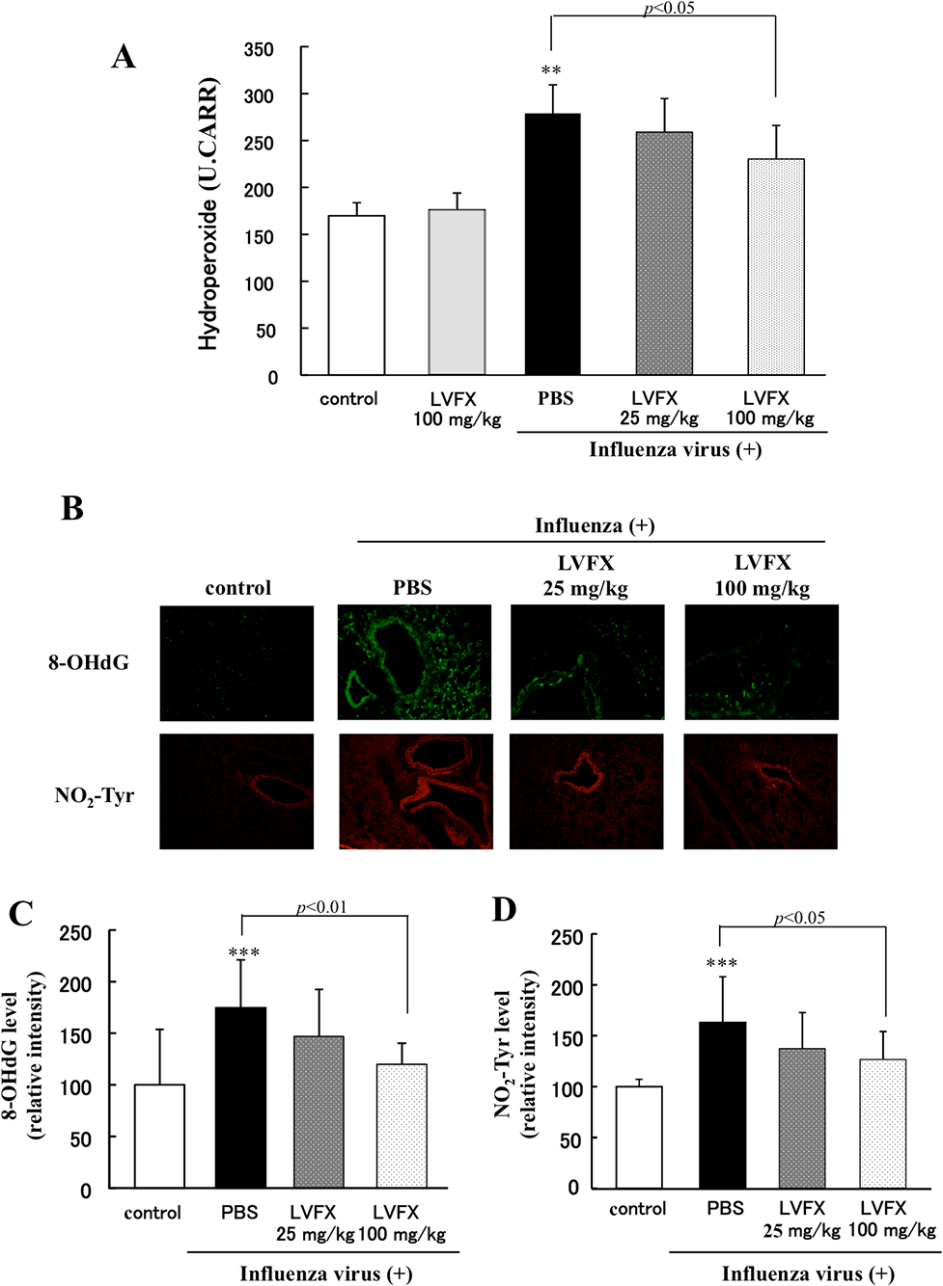
The effect of LVFX on oxidative stress in influenza virus-infected mice. The effect of LVFX on the accumulation of oxidative stress in the blood or lungs were determined (A) by measuring hydroperoxide levels (dROMs test) or (B) immunostaining for 8-OHdG (upper panel) and NO_2_-Tyr (lower panel) level at the day 7 after influenza virus infection. The fluorescence intensity of (C) 8-OHdG and (D) NO_2_-Tyr were quantified by the imageJ software. Each bar represents the mean ± SD (n = 5–9). **p<0.01, ***p<0.001 vs control.

The findings reported herein confirm that LVFX exerts an anti-oxidative effect in an influenza virus infected mouse model. We used the dROMs test to evaluate the degree of oxidative stress because it has been reported that the level of hydroperoxide radicals, as measured by a dROMs test, was significantly increased in influenza virus infected mice [12]. As shown in Fig 4A, at day 7 after infection with the influenza virus, the level of hydroperoxide radicals in the blood was significantly increased, but was significantly lowered by the administration of LVFX in a dose-dependent manner.

In addition to ROS, NO production is also induced various virus infections, including influenza. Thus, we further examined the effect of LVFX on oxidative and nitrative stress in the lungs of influenza virus infected mice. In this experiment, changes in oxidative and nitrative stress were evaluated by the accumulation of 8-OHdG and NO_2_-Tyr, reactive metabolites of the peroxynitrite anion that is produced by the rapid reaction of NO and the superoxide anion, respectively. As shown in Fig 4B–4D the levels of both 8-OHdG (upper panel) and NO_2_-Tyr (lower panel) in lung tissue on day 7 were markedly increased as the result of the influenza virus infection (vehicle (i.p)), while the LVFX treatment clearly decreased the levels of these oxidative and nitrative markers.

### Effect of LVFX on NOx and IFN-γ levels in BALF from influenza virus-infected mice

**Fig 5:**
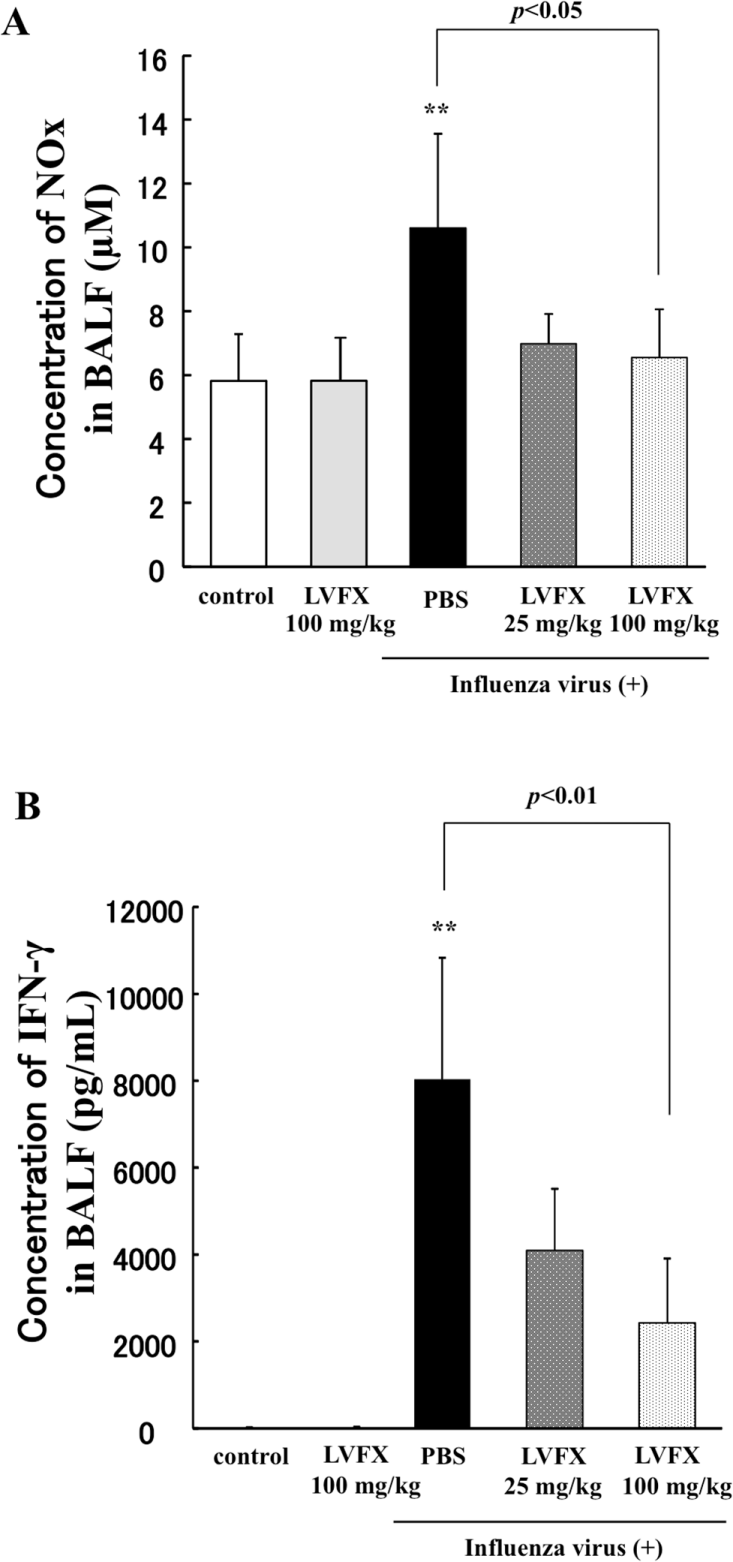
The effect of LVFX on NO and IFN-γ production in BALF of influenza virus-infected mice. (A) The effect of LVFX on the accumulation of NO in BALF was determined by measuring NOx (NO_2_^-^ and NO_3_^-^). (B) IFN-γ levels in the BALF were determined by ELISA on day 7 after influenza virus administration. Each bar represents the mean ± SD (n = 4–5). *p<0.05, **p<0.01 vs control.

Previous studies demonstrated that an overproduction of NO is induced by the elevation of IFN-γ levels in influenza virus infections [11, 24]. In addition, it was also reported that erythromycin can be of therapeutic value for influenza virus induced pneumonia, by inhibiting IFN-γ production and reducing the overproduction of NO in the lungs [24]. To clarify the mechanism responsible for the inhibition of lung damage by LVFX, we examined the effect of LVFX on the level of both NOx (the sum of NO metabolites, NO_2_^-^/NO_3_^-^) and IFN-γ in BALF. Consistent with results reported in a previous study [24], the levels of both NOx and IFN-γ in BALF were markedly increased on day 7 after the influenza virus infection in our model (Fig 5A and 5B). In such a circumstance, the administration of LVFX significantly suppressed the elevation of both NOx and IFN-γ levels. We also measured the decrease in the concentration of TNF-α in BALF by the LVFX administration compared with the control. However, no differences were found between a LVFX dose of 25 mg/kg and a dose of 100 mg/kg (S5 Fig).

## Discussion

Recently reported experimental and clinical findings indicate that neutrophil-derived ROS and RNS play an important role in influenza virus-induced lung injuries and subsequent mortality [9–11]. Thus, anti-oxidative or anti-nitrative therapy represents a new strategy for combating influenza and related diseases. The findings reported herein show that LVFX has potent anti-oxidative and anti-nitrative properties that lessen the effects caused by a lung injury and consequently, improved the survival of mice that had been infected with the H1N1 influenza virus without changing the virus titer.

It has been reported that FQs possess anti-oxidative and anti-nitrative properties that are independent of their anti-microbial activity. For example, Akamatsu et al. reported that ofloxacin, a racemic mixture of LVFX, showed inhibitory activity against neutrophil-derived ROS (O_2_^∙-^, OH radical, H_2_O_2_) [18]. However, most of these studies were limited to *in vitro* assessments and only a few attempts to demonstrate the anti-oxidative activity of FQ *in vivo* as well as attempts to compare the activity between FQs have been reported. Among the FQs, LVFX was found to exhibit potent anti-oxidative properties both *in vitro* and *in vivo*. Moreover, it is also important to clarify which specific ROS is scavenged by LVFX. ESR spin-trapping can be used, not only to clarify the magnitude of radical scavenging activity, but also to identify which radical species is eliminated. In this study, we used DMPO as a spin-trapping agent because it produces signals that are unique for different types of ROS, when an adduct is formed [25]. For example, four highly characteristic signals (the ratio of each signal height = 1:2:2:1) corresponding to an adduct DMPO and a OH radical (DMPO-OH) are produced. As previously reported, these four typical signals are produced in a H_2_O_2_/UV system, indicating that OH radicals are generated. This was further confirmed by the dramatic reduction in the DMPO-OH adduct signal in the presence of the specific OH scavenger (dimethylthiourea: DMTU), but not by a specific superoxide scavenger (superoxide dismutase: SOD) (S6 Fig). A similar inhibition in a H_2_O_2_/UV system and PMA-stimulated neutrophils was observed in the case of LVFX, indicating that LVFX is capable of scavenging OH radicals. In contrast to the OH radical, LVFX did not scavenge O_2_^∙-^ radicals that were produced in a Xanthine/Xanthine Oxidase system (data not shown). These findings are consistent with the action of ofloxacin. Thus, such a potent OH radical scavenging activity of LVFX could contribute to a reduction in the extent of lung injuries and an improved survival of the influenza virus infected mice.

In the present study, an LVFX dosage of 200 μM was sufficient to inhibit the ESR signal in the H_2_O_2_/UV system, while 1000 μM was required in the case of a neutrophil derived ROS detection system. Such an inconsistency could be explained by the relationship between DMPO and the anti-oxidant as follows;[26]

k_2_ = k_1_[DMPO]/IC_50_

k_2_: rate constant for reaction with ROS and DMPO

k_1_: rate constant for reaction with ROS and anti-oxidant

IC_50_: half maximal inhibitory concentration of anti-oxidant

Here, to detect a sufficient ESR signal in the neutrophil derived ROS detection system, we used a DMPO concentration that is five times higher than that used in the H_2_O_2_/UV system. According to the above relationship, five times higher concentration of LVFX would be needed in the experiment of neutrophil system.

In addition to oxidative stress, nitrative stress is also involved in influenza virus-induced lung injuries and mortality [7, 11, 24]. Since the increased production of NO is heavily dependent on the expression of iNOS which, in turn, is induced by IFN-γ, the presence of a NO synthase inhibitor or the suppression of excessive levels of IFN-γ could result in the survival of more of the mice [11, 24]. Moreover, compared with wild-type mice, in extracellular SOD transgenic mice, not only IFN-γ but also NOx levels and lung nitrotyrosine formation induced by a influenza virus infection are inhibited[10]. Therefore, it is conceivable that NO or NO-derived species act to enhance influenza-associated pathology. This notion is supported in the literature based on the use of NOS inhibitors during infections with murine cytomegalovirus [27]. These findings point to the importance of the role of the ROS-IFN-γ-NO system in lung injuries in mice that were infected with the influenza virus.

The IFN-γ that is produced by the influenza virus infection model mice is derived from T cells [28, 29]. Kaminski et al. reported that ciprofloxacin, a fluoroquinolone antibiotic, exerts an immunosuppressive effect on human T cells by depleting mtDNA, impairing mitochondrial function, thus resulting in a reduced ROS generation. They also indicated that H_2_O_2_-mediated oxidative signals control this gene transcription [30]. Moreover, an SOD mimic inhibits antigen-presenting cell dependent T cell proliferation and IFN-γ production. Akamatsu et al. reported that ofloxacin exerts an inhibitory effect against O_2_^∙-^ derived from neutrophils that had been stimulated by a zymosan treatment. In addition, deferoxamine (an iron chelator) and DMTU have been reported to inhibit the inflammatory response of endothelial cells by decreasing the levels of NF-κB, a regulatory molecule for IL-1β, TNF-α or IFN-γ [31, 32]. These findings suggest that the reduction in IFN-γ caused by the administration of LVFX is partly dependent on its anti-oxidative effect. On the other hand, IFN-γ derived from cytotoxic CD8 T cells (Tc) is also important for achieving this amelioration, but Tc1 and Tc2 play different roles [33]. Our findings are different from those reported for KO mice, in which LVFX was reported to not completely suppress IFN-γ production. The over expression of SOD or an erythromycin treatment has been reported to partially suppress IFN-γ production (half ~ one third), which ameliorated influenza virus infections [10, 24]. Susceptibility to bacterial pneumonia is enhanced in influenza virus infections [14, 34], and the mechanism responsible for this appears to involve the production of excess IFN-γ [35]. From the standpoint of inhibiting secondary bacterial infections, the suppression of IFN-γ leads to the restoration of innate immunity against pneumonia.

Other mechanisms for regulating the immune system by FQs are known [17]. Cyclic AMP, protein kinase A and Phosphodiesterases (PDE), signal molecules or enzymes associated with a series of intracellular protein phosphorylation or transcription factor activation/suppression, are known to be regulated by FQs. The inhibitory effect on TNF-α production triggered by CPFX is mediated by PDE, leading to the accumulation of cAMP. Therefore, other inhibitory effects of FQs may exist in the inflammatory response under conditions of an influenza virus infection.

In the present study, we found that LVFX effectively suppressed nitrative stress levels in the lung and BALF, as evidenced by the immnofluorostaining of NO_2_-Tyr and the concentration of NOx, indicating that the effect of LVFX on survival or a lung injury partly depends on the inhibition of nitrative stress as well as oxidative stress. However, other mechanisms and more extensive research such as over-exuberant immune responses are needed.

## Conclusion

The results reported herein show that a therapeutic dose of LVFX significantly inhibits the lethal effects of influenza virus-induced lung injury in mice, partly via its anti-oxidative and NO regulatory effects. Interestingly, these effects appear to be independent of its antibacterial properties. Thus, such a pleiotropic effect of LVFX has the potential to be of great benefit in the clinical treatment of influenza virus-induced pneumonia preceding event of secondary infections by bacteria.

## Supporting Information

S1 Fig The effect of chloral hydrate on systemic oxidative stress. The effect of chloral hydrate on oxidative stress was determined by measuring the hydroperoxide level in serum. Mice were anesthetized with or without chloral hydrate (500 mg/kg. i.p), and blood was collected at day 1 and 7. Serum hydroperoxide level was determined by dROMs test. Each bar represents the mean ± SD (n = 3).
(DOCX)

S2 Fig The scheme of experimental protocol for the effective evaluation of LVFX on influenza virus-infected mice. Mice were infected with PR8 on day 0, and then they were administrated with LVFX (25, 100 mg/kg) for 5 days from day 2. Efficacy and survival were evaluated on day 7 or 14, respectively.
(DOCX)

S3 Fig The effect of LVFX on body weight loss during influenza virus infection. The effect of LVFX on body weight (BW) change during influenza virus infection was monitored. Mice were infected with influenza virus and administrated (A) PBS (n = 10), (B) LVFX 25 mg/kg/day (n = 10) or (C) LVFX 100 mg/kg/day (n = 10). BW decreasing rate (%) was calculated with follow: BW decreasing rate (%) = (BW–day 0 BW)/day 0 BW x 100; >30% decrease considered dead. Experiment was repeated three times.
(DOCX)

S4 Fig The effect of LVFX on pulmonary damage in influenza virus-induced mice. Section of lung tissue were prepared at the day 7 after influenza virus infection, and subjected to histopathological examination with HE staining. All lung sections are shown in upper figure and high magnification images (x40) are shown lower.
(DOCX)

S5 Fig The effect of LVFX on TNF-αproduction in BALF of influenza virus-infected mice. The level of TNF-α level in BALF was determined by ELISA on day 7 after the administration of the influenza virus. Each bar represents the mean ± SD (n = 4–5). **p<0.01 vs control.
(DOCX)

S6 Fig The effect of OH radicals specific scavenger on irradiation of UV to H_2_O_2_ system. The effect of specific scavengers of OH radicals (dimethylthiourea: DMTU) on UV/H_2_O_2_ system was evaluated. (A) The reaction mixtures, which contained 500 μM H_2_O_2_, 100 μM DTPA and 4.5 mM DMPO, were incubated with or without DMTU or superoxide dismutase (SOD), and immediately transferred to a ESR flat cell and irradiated at 254 nm for 30 s. After UV-irradiation, the ESR flat cells immediately placed in a JES-TE 200 ESR spectrometer. ESR spectrum of DMPO spin adducts and (B) the quantitation of the concentration of OH radicals is shown.
(DOCX)
