# Supplementary material for: Pleiotropic Effects of Levofloxacin, Fluoroquinolone Antibiotics, against Influenza Virus-Induced Lung Injury
Source: PLoS One. 2015 Jun 18;10(6):e0130248. doi: 10.1371/journal.pone.0130248 (PMC4473075; doi:10.1371/journal.pone.0130248)
Supplement: S1 Fig — The effect of chloral hydrate on oxidative stress was determined by measuring the hydroperoxide level in serum. Mice were anesthetized with or without chloral hydrate (500 mg/kg. i.p), and blood was collected at day 1 and 7. Serum hydroperoxide level was determined by dROMs test. Each bar represents the mean ± SD (n = 3). (DOCX) [file pone.0130248.s001.docx]

**Supporting Information**

**Pleiotropic effects of levofloxacin, fluoroquinolone antibiotics, against influenza virus-induced lung injury**

Yuki Enoki, Yu Ishima, Ryota Tanaka, Keizo Sato, Kazuhiko Kimachi, Tatsuya Shirai, Hiroshi Watanabe, Victor T. G. Chuang, Yukio Fujiwara, Motohiro Takeya, Masaki Otagiri, Toru Maruyama

**SUPPORTING FIGURE**

**S1_Fig.**

**S1_Fig. The effect of chloral hydrate on systemic oxidative stress.**

The effect of chloral hydrate on oxidative stress was determined by measuring the hydroperoxide level in serum. Mice were anesthetized with or without chloral hydrate (500 mg/kg. i.p), and blood was collected at day 1 and 7. Serum hydroperoxide level was determined by dROMs test. Each bar represents the mean±SD (n = 3).
