# Supplementary material for: Pleiotropic Effects of Levofloxacin, Fluoroquinolone Antibiotics, against Influenza Virus-Induced Lung Injury
Source: PLoS One. 2015 Jun 18;10(6):e0130248. doi: 10.1371/journal.pone.0130248 (PMC4473075; doi:10.1371/journal.pone.0130248)
Supplement: S2 Fig — Mice were infected with PR8 on day 0, and then they were administrated with LVFX (25, 100 mg/kg) for 5 days from day 2. Efficacy and survival were evaluated on day 7 or 14, respectively. (DOCX) [file pone.0130248.s002.docx]

**Supporting Information**

**Pleiotropic effects of levofloxacin, fluoroquinolone antibiotics, against influenza virus-induced lung injury**

Yuki Enoki, Yu Ishima, Ryota Tanaka, Keizo Sato, Kazuhiko Kimachi, Tatsuya Shirai, Hiroshi Watanabe, Victor T. G. Chuang, Yukio Fujiwara, Motohiro Takeya, Masaki Otagiri, Toru Maruyama

**SUPPORTING FIGURE**

**S2_Fig.**

**S2_Fig. The scheme of experimental protocol for the effective evaluation of LVFX on influenza virus-infected mice.**

Mice were infected with PR8 on day 0, and then they were administrated with LVFX (25, 100 mg/kg) for 5 days from day 2. Efficacy and survival were evaluated on day 7 or 14 respectively.
