# Supplementary material for: Pleiotropic Effects of Levofloxacin, Fluoroquinolone Antibiotics, against Influenza Virus-Induced Lung Injury
Source: PLoS One. 2015 Jun 18;10(6):e0130248. doi: 10.1371/journal.pone.0130248 (PMC4473075; doi:10.1371/journal.pone.0130248)
Supplement: S3 Fig — The effect of LVFX on body weight (BW) change during influenza virus infection was monitored. Mice were infected with influenza virus and administrated (A) PBS (n = 10), (B) LVFX 25 mg/kg/day (n = 10) or (C) LVFX 100 mg/kg/day (n = 10). BW decreasing rate (%) was calculated with follow: BW decreasing rate (%) = (BW–day 0 BW)/day 0 BW x 100; >30% decrease considered dead. Experiment was repeated three times. (DOCX) [file pone.0130248.s003.docx]

**Supporting Information**

**Pleiotropic effects of levofloxacin, fluoroquinolone antibiotics, against influenza virus-induced lung injury**

Yuki Enoki, Yu Ishima, Ryota Tanaka, Keizo Sato, Kazuhiko Kimachi, Tatsuya Shirai, Hiroshi Watanabe, Victor T. G. Chuang, Yukio Fujiwara, Motohiro Takeya, Masaki Otagiri, Toru Maruyama

**SUPPORTING FIGURE**

**S3_Fig.**

**S3_Fig. The effect of LVFX on body weight loss during influenza virus infection.**

The effect of LVFX on body weight (BW) change during influenza virus infection was monitored. Mice were infected with influenza virus and administrated (A) PBS (n=10), (B) LVFX 25 mg/kg/day (n=10) or (C) LVFX 100 mg/kg/day (n=10). BW decreasing rate (%) was calculated with follow:

BW decreasing rate (%) = (BW – day 0 BW)/day 0 BW x 100

>30% decrease considered dead. Experiment was repeated three times.
