# Supplementary material for: Pleiotropic Effects of Levofloxacin, Fluoroquinolone Antibiotics, against Influenza Virus-Induced Lung Injury
Source: PLoS One. 2015 Jun 18;10(6):e0130248. doi: 10.1371/journal.pone.0130248 (PMC4473075; doi:10.1371/journal.pone.0130248)
Supplement: S4 Fig — Section of lung tissue were prepared at the day 7 after influenza virus infection, and subjected to histopathological examination with HE staining. All lung sections are shown in upper figure and high magnification images (x40) are shown lower. (DOCX) [file pone.0130248.s004.docx]

**Supporting Information**

**Pleiotropic effects of levofloxacin, fluoroquinolone antibiotics, against influenza virus-induced lung injury**

Yuki Enoki, Yu Ishima, Ryota Tanaka, Keizo Sato, Kazuhiko Kimachi, Tatsuya Shirai, Hiroshi Watanabe, Victor T. G. Chuang, Yukio Fujiwara, Motohiro Takeya, Masaki Otagiri, Toru Maruyama

**SUPPORTING FIGURE**

**S4_Fig.**

**Control**

**Control + LVFX 100 mg/kg**

**Influenza + PBS**

**Influenza + LVFX 25 mg/kg**

****  **Influenza + LVFX 100 mg/kg**

**S4_Fig. The effect of LVFX on pulmonary damage in influenza virus-induced mice.**

Section of lung tissue were prepared at the day 7 after influenza virus infection, and subjected to histopathological examination with HE staining. All lung sections are shown in upper figure and high magnification images (x40) are shown lower.
