# Supplementary material for: Pleiotropic Effects of Levofloxacin, Fluoroquinolone Antibiotics, against Influenza Virus-Induced Lung Injury
Source: PLoS One. 2015 Jun 18;10(6):e0130248. doi: 10.1371/journal.pone.0130248 (PMC4473075; doi:10.1371/journal.pone.0130248)
Supplement: S5 Fig — The level of TNF-α level in BALF was determined by ELISA on day 7 after the administration of the influenza virus. Each bar represents the mean ± SD (n = 4–5). **p<0.01 vs control. (DOCX) [file pone.0130248.s005.docx]

**Supporting Information**

**Pleiotropic effects of levofloxacin, fluoroquinolone antibiotics, against influenza virus-induced lung injury**

Yuki Enoki, Yu Ishima, Ryota Tanaka, Keizo Sato, Kazuhiko Kimachi, Tatsuya Shirai, Hiroshi Watanabe, Victor T. G. Chuang, Yukio Fujiwara, Motohiro Takeya, Masaki Otagiri, Toru Maruyama

**SUPPORTING FIGURE**

**S5_Fig.**

**S5_Fig. The effect of LVFX on TNF-α production in BALF of influenza virus-infected mice.**

The level of TNF-α level in BALF was determined by ELISA on day 7 after the administration of the influenza virus. Each bar represents the mean±SD (n = 4-5). **p<0.01 vs control.
