# Supplementary material for: Pleiotropic Effects of Levofloxacin, Fluoroquinolone Antibiotics, against Influenza Virus-Induced Lung Injury
Source: PLoS One. 2015 Jun 18;10(6):e0130248. doi: 10.1371/journal.pone.0130248 (PMC4473075; doi:10.1371/journal.pone.0130248)
Supplement: S6 Fig — The effect of specific scavengers of OH radicals (dimethylthiourea: DMTU) on UV/H2O2 system was evaluated. (A) The reaction mixtures, which contained 500 μM H2O2, 100 μM DTPA and 4.5 mM DMPO, were incubated with or without DMTU or superoxide dismutase (SOD), and immediately transferred to a ESR flat cell and irradiated at 254 nm for 30 s. After UV-irradiation, the ESR flat cells immediately placed in a JES-TE 200 ESR spectrometer. ESR spectrum of DMPO spin adducts and (B) the quantitation of the concentration of OH radicals is shown. (DOCX) [file pone.0130248.s006.docx]

**Supporting Information**

**Pleiotropic effects of levofloxacin, fluoroquinolone antibiotics, against influenza virus-induced lung injury**

Yuki Enoki, Yu Ishima, Ryota Tanaka, Keizo Sato, Kazuhiko Kimachi, Tatsuya Shirai, Hiroshi Watanabe, Victor T. G. Chuang, Yukio Fujiwara, Motohiro Takeya, Masaki Otagiri, Toru Maruyama

**SUPPORTING FIGURE**

**S6_Fig.**

**S6_Fig. The effect of OH radicals specific scavenger on irradiation of UV to H2O2 system.**

The effect of specific scavengers of OH radicals (dimethylthiourea: DMTU) on UV/H2O2 system was evaluated. (A) The reaction mixtures, which contained 500 μM H2O2, 100 μM DTPA and 4.5 mM DMPO, were incubated with or without DMTU or superoxide dismutase (SOD), and immediately transferred to a ESR flat cell and irradiated at 254 nm for 30 s. After UV-irradiation, the ESR flat cells immediately placed in a JES-TE 200 ESR spectrometer. ESR spectrum of DMPO spin adducts and (B) the quantitation of the concentration of OH radicals is shown.
